# Expenses related to hospital admissions for the elderly in Brazil: perspectives of a decade

**DOI:** 10.1590/S1679-45082013000400019

**Published:** 2013

**Authors:** Rodrigo Eurípedes da Silveira, Álvaro da Silva Santos, Mariana Campos de Sousa, Taciana Silva Alves Monteiro

**Affiliations:** 1 Universidade Federal do Triângulo Mineiro, Uberaba, MG, Brazil

**Keywords:** Health expenditures, Hospitalization, Aged, Epidemiology, Health of the elderly

## Abstract

**Objective:**

To describe the profile of morbidities and expenses related to hospitalization of the elderly compared to the adult population (20 to 59 years).

**Methods:**

A descriptive, cross-sectional investigation of hospitalizations of the elderly (60 years or older) in Brazil during the period from 2002 to 2011, with data from DATASUS and based on ICD-10.

**Results:**

Although the highest number of hospitalizations were in the adult age range, the expenses were greater with the elderly, and in this case especially with mental and behavioral disorders, musculoskeletal and connective tissue diseases, followed by circulatory diseases and external causes.

**Conclusion:**

The results suggest the adoption of more comprehensive policies and increased investment in health promotion, disease prevention, and appropriate and suitable treatment for the most prevalent diseases in the elderly, particularly in primary care.

## RESUMO

**Objetivo:**

Descrever o perfil de morbidades e os gastos relacionados a internações de idosos comparados aos da população de adultos (20 a 59 anos).

**Métodos:**

Trata-se de uma pesquisa descritiva, transversal, sobre as internações hospitalares de idosos (com 60 anos ou mais de idade) no Brasil no período de 2002 a 2011, com dados do DATASUS e com base na CID-10.

**Resultados:**

Embora o maior número de internações esteja na faixa etária adulta, a proporção de gastos foi maior com os idosos e, neste caso, em especial com doenças mentais e comportamentais, osteomusculares e do tecido conjuntivo, seguidas das doenças do aparelho circulatório e causas externas.

**Conclusão:**

Os resultados sugerem a adoção de políticas mais abrangentes e maior investimento em ações de promoção da saúde, prevenção de agravos e tratamento oportuno e adequado para as doenças mais prevalentes nos idosos, em especial na Atenção Primária à Saúde.

## INTRODUCTION

In a current context of health sciences, similar to various developing countries, Brazil is rapidly aging. This is a reflection of a process called demographic transition, resulting from the reduction in mortality and fertility rates^(1)^. However, in addition to these demographic changes, there are simultaneous alterations in the epidemiological profile, both in morbidity and mortality of the population, especially due to reduced incidence of infectious parasitic diseases and increase in non-communicable diseases^(2)^.

It is estimated that the population over 60 years in age will go from less than 20 million in 2010, to approximately 65 million in 2050^(3)^. As a consequence of this change in age profile of the population, greater fiscal pressures are expected on public healthcare services and social security, generating a great demand for health services which will in turn lead to an increase in expenditures with medical costs and hospital admissions^(4)^.

If, on the one hand the elderly display a greater burden of diseases and disabilities, and therefore, use a large portion of the health services, on the other hand, inefficiency and high costs related to the current models of elderly healthcare stand out^(3,5)^. In this way, an outline of the profile and statistics of hospitalization rates of the elderly may serve as a model for drawing up more effective strategies of health promotion and disease prevention.

In this regard, the Unified Healthcare System Hospital Information Services (SIH-SUS, acronym in Portuguese) provides a database that accounts for 80% of hospital admissions in the country, enabling not only profiling of hospitalizations, but also allowing an assessment of their economic impact^(6)^.

## OBJECTIVE

Based on questions as to the true proportions of hospitalization costs of the elderly in Brazil, this investigation had the objective of describing the profile of morbidities and expenses related to hospital admissions of the elderly compared to those of the adult population (20 to 59 years).

## METHODS

A descriptive cross-sectional study of hospital admissions of the elderly (60 years or more) in Brazil during the period from 2002 to 2011.This population was grouped into three categories: 60 to 69 years, 70 to 79, and 80 years or more, considering the stages specified by Fletcher & Fletcher^(7)^. For comparison purposes, the adult age range was also presented, considered from 20 to 59 years.

The resident population during the period (2002 to 2011), used to calculate some of the coefficients, was made available by the SUS Information Technology Department (DATASUS)^(8)^. The run chart studied is based on official and secondary data on hospital admissions obtained from the SIH-SUS. The variables analyzed were gender, age, type of Hospital Admission Authorization (AIH, acronym in Portuguese), cost of hospitalization, days of stay, and working diagnosis, as per chapters of the International Classification of Diseases and Health-Related Problems – Tenth Revision (ICD-10). For the working diagnosis, all the chapters of ICD-10 were used except Chapter XV, which refers to admissions for natural deliveries and caesarean sections (codes 080 to 084.9), maintaining disorders related to pregnancy and puerperium.

The major groups of causes were considered, as well as diagnoses due to selected causes including circulatory system diseases; respiratory system diseases; digestive system diseases; infectious and parasitic diseases; genitourinary diseases; external causes; endocrine, nutritional, and metabolic diseases; nervous system diseases; mental and behavioral disorders; neoplasms; musculoskeletal system and connective tissue diseases; and other diseases. This list was partially set up, for comparison purposes, based on the major causes of hospital admissions among the elderly Brazilian population^(4)^ and the primary causes of hospital admissions among elderly Americans^(3)^.

The analyses were performed based on absolute numbers, percentages, and a few indicators, namely: 
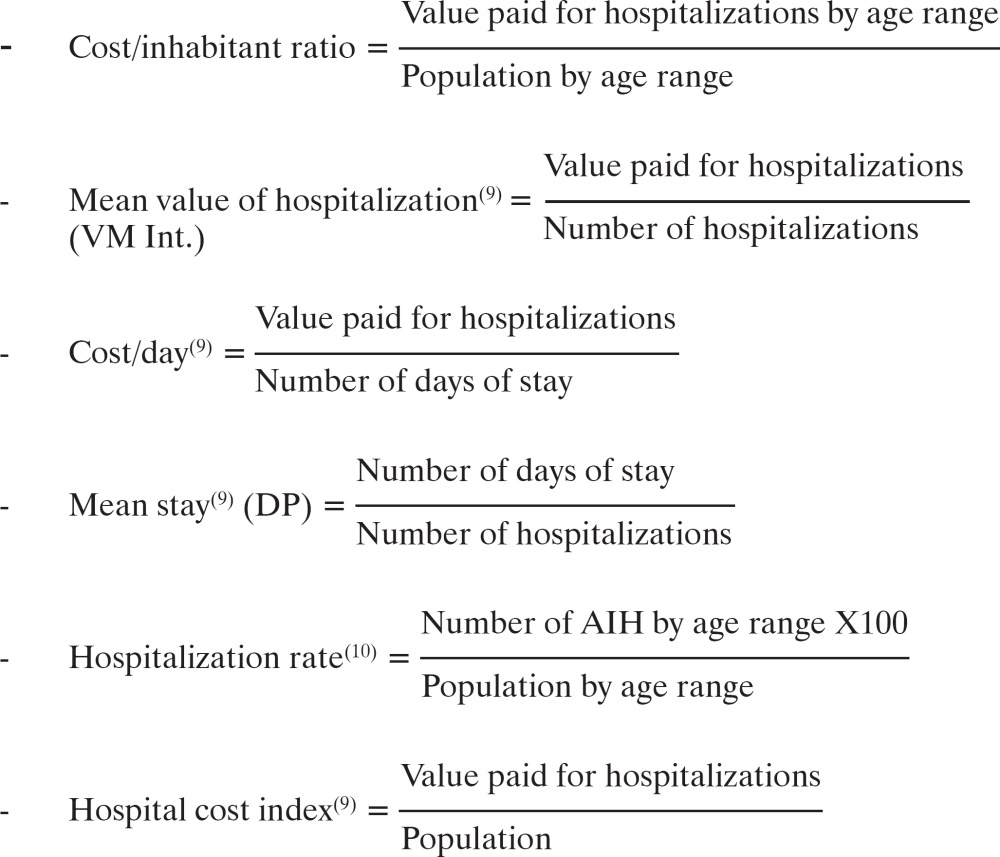



The data from hospitalization records are not subject to sampling error, since they cover the totality of events occurred. However, they suffer the effects of random error in the registration process and population estimate. Since it is a study that uses secondary data from an electronic database that makes such information available for access to the general public (DATASUS), this investigation was not submitted to a Committee on Research Ethics in Human Beings. The development of this investigation occurred during the period from August 2002 to December 2012.

## RESULTS

During the 10 years considered in this study, there were 20,590,599 hospital admissions among elderly Brazilians within the scope of SUS. Of these hospitalizations, 11,434,487 (55.5%) corresponded to the female gender, and the sum of values paid for all these hospitalizations was R$ 21,545,274,041, as displayed on table 1.

**Table 1:** Distribution of inhabitants, hospital admissions, value paid, and cost/inhabitant ratio within the scope of the Unified Healthcare System. Brazil, 2002–2011

Age range (years)		Inhabitants*		Hospitalizations		Value paid (R$)		Cost/inhabitant ratio (R$)
		n	%	n	%	n	%	
Men								
	20–59	52,325,466	85.11	19,718,102	63.33	10,122,183,665	47.08	193.45
	60–69	5,265,099	8.56	5,003,499	16.07	5,467,634,284	25.43	1,038.47
	70–79	2,757,891	4.49	4,136,869	13.29	4,051,005,707	18.84	1,468.88
	80+	1,133,122	1.84	2,274,661	7.31	1,857,720,080	8.64	1,639.47
	60+**	9,156,112	14.89	11,415,029	36.67	11,376,360,071	52.92	1,242.49
Subtotal		61,481,578	100.00	31,133,131	100.00	21,498,543,736	100.00	349.67
Women								
	20–59	54,916,569	82.77	39,630,866	77.52	27,407,265,524	72.94	499.07
	60–69	6,084,830	9.17	4.577.541	8.95	4,210,829,863	11.21	692.02
	70–79	3,547,194	5.35	4,117,805	8.05	3,674,380,784	9.78	1,035.86
	80+	1,802,463	2.72	2,798,079	5.47	2,283,703,323	6.08	1,266.99
	60+**	11,434,487	17.23	11,493,425	22.48	10,168,913,970	27.06	889.32
Subtotal		66,351,056	100.00	51,124,291	100.00	37,576,179,494	100.00	566.32
General population (men+women)								
	20–59	107,242,035	83.89	59,348,968	72.15	37,529,449,189	63.53	349.95
	60–69	11,349,929	8.88	9,581,040	11.65	9,678,464,147	16.38	852.73
	70–79	6,305,085	4.93	8,254,674	10.04	7,725,386,491	13.08	1,225.26
	80+	2,935,585	2.30	5,072,740	6.17	4,141,423,403	7.01	1,410.77
	**Subtotal of 60+	20,590,599	16.11	22,908,454	27.85	21,545,274,041	36.47	1,046.36
Total		127,832,634	100.00	82,257,422	100.00	59,074,723,230	100.00	462.13

* Value verified by 2010 Census (IBGE);

** subtotal refers to the sum of persons aged 60 years and more.

Source: DATASUS, 2012^(8)^.

The elderly represented 16.11% during the period, contributing towards 27.85% of hospitalizations and 36.47% of the resources paid for them. The ratio between the percentage of values paid for hospitalizations and the percentage of the population increases gradually with age in men and women. This ratio was equal to 3.55 for elderly men, 1.57 for elderly women, and 2.26 for the elderly of both genders. When these data are divided by age range, the cost/inhabitant ratio increases significantly with age: 0.76 in the range of 20–59 years; 1.85 in 60–69; 2.65 in 70–79; and 3.05 in the range of 80+ years.

Still on table 1, one can note a ratio of costs of around eight times more expensive for the male elderly population (R$ 1,639.47) relative to the adult age range (R$ 193.45). As to women, the proportion is up to 2.5 times more expensive for hospitalization of the elderly woman over 80 years of age (R$ 1,266.99) relative to the adult woman (R$ 499.07).

Table 2 shows the mean value of hospitalization, the daily cost, and how much time that elderly individual remained hospitalized. The costs of hospitalization for adult and elderly men were similar, while for women, the mean hospital stay cost was R$ 190.00 more for the elderly. Between the genders, there is distancing four times greater of the values of the adult population, and the most expensive mean hospitalization cost is for the male gender. This tendency is inverted in the assessment of cost/day for the adult population, with values close to those for the elderly (R$ 104.27 and R$ 100.30). Adult men remain in the hospital twice as long while among the elderly, hospital length of stay is similar.

**Table 2:** Distribution of the mean value of hospitalization, cost/day, and mean length of stay, by gender and region of the country. Brazil, 2002–2011

		Mean value of hospitalization (R$)		Cost/day (R$)		Mean length of stay	
		20–59	60+	20–59	60+	20–59	60+
Gender							
	Male	766.76	766.12	76.53	104.27	10.02	7.4
	Female	525.75	710.11	103.08	100.3	5.4	7.1
Region							
	Southeast	692.15	855.58	83.82	97.21	8.4	8.9
	South	656.53	775.32	103.07	125.64	6.3	6.3
	Northeast	515.98	584.08	86.69	95.07	6.3	6.2
	North	418.58	521.41	103.81	88.48	4.4	5.9
	Center-West	529.37	628.99	94.44	111.19	5.6	5.8

Source: DATASUS, 2012^(8)^.

As to regions, the Southeast Region showed higher mean values than the rest. The cost/day of hospitalization is the highest in the North (R$ 103.81) and South (R$ 103.07) Regions, and the latter one represents the highest daily values among the elderly (R$ 125.64). Both adults and the elderly remain in the hospital longer in the Southeast Region (8.9 days for the elderly individual and 8.4 days for the adult), while in the North Region the values are 5.9 days for the elderly and 4.4 days for adults.

Table 3 shows the calculations of hospitalizations as to this group of causes, extracted from chapters of the ICD-10. Among the adult population, hospitalizations with higher values are those caused by mental and behavioral disorders, followed by nervous system and circulatory system diseases. For the elderly population, the value of hospitalization due to mental diseases is higher and twice that manifested between 20 and 59 years; other more expensive causes (musculoskeletal and circulatory diseases) have similar values.

**Table 3:** Distribution of mean expense, cost/day, and mean hospital length of stay by age range. Brazil, 2002–2011

ICD-10 chapter	Number of hospitalizations		Mean value of hospitalization (R$)		Cost/day (R$)		Mean length of stay	
	20–59	60+	20–59	60+	20–59	60+	20–59	60+
Mental and behavioral disorders	2,649,217	209,003	1,559.01	3,324.19	33.06	32.28	47.16	102.99
Nervous system diseases	799,625	423,209	1,453.85	1,109.34	82.28	90.51	17.67	12.26
Musculoskeletal and connective tissue diseases	1,475,798	523,937	880.71	1,399.64	173.15	164.63	5.09	8.51
External causes	4,739,648	1,233,313	829.28	1,229.66	158.21	185.28	5.24	7.67
Neoplasms	3,404,143	1,841,718	943.84	1,120.31	177.51	161.85	5.32	6.92
Circulatory system diseases	5,025,540	6,414,549	1,304.23	1,346.05	228.33	197.39	5.71	6.82
Infectious and parasitic diseases	3,304,970	1,703,416	542.12	717.33	75.97	107.31	7.14	6.68
Respiratory system diseases	3,696,055	4,025,822	606.22	667.19	113.38	109.09	5.35	6.12
Endocrine, nutritional, and metabolic diseases	1,104,244	1,190,170	519.75	387.06	87.59	63.64	5.93	6.08
Genitourinary system diseases	4,787,674	1,448,201	457.83	520.36	131.04	94.84	3.49	5.49
Digestive system diseases	5,649,147	2,329,760	556.18	615.25	138.36	121.57	4.02	5.06
Other causes	22,713,097	1,565,512	877.55	1,130.58	127.17	120.76	10.19	15.87

Source: DATASUS, 2012^(8)^.

As can be seen, the daily values of hospitalizations show a similar distribution among adults and the elderly, especially for circulatory system problems. In the elderly population, external causes have a higher value than in the other age groups observed. As to length of stay, in general, those aged over 60 years spend more time hospitalized, except for infectious diseases and nervous system disorders. It is noteworthy mentioning that the length of stay for mental and behavioral disorders reaches 103 days among the elderly (Table 3).

The hospitalization rate (Table 4) has a contrasting distribution between men and women, despite the fact that, in general, they maintain increasing values in the elderly. The hospitalization rates for elderly men - due to all causes - are higher than those for women; except for endocrine, nutritional, and metabolic diseases; and musculoskeletal system and connective tissue diseases.

**Table 4:** Distribution of hospital admission rates and indexes of cost by gender and age range. Brazil, 2002–2011

ICD-10 chapter	Hospital admission rate				Cost index (R$/person)			
	Male		Female		Male		Female	
	20–59	60+	20–59	60+	20–59	60+	20–59	60+
Circulatory system diseases	5.17	45.01	5.77	37.92	6.51	52.49	5.06	36.09
Respiratory system diseases	4.11	29.65	4.07	22.8	2.02	14.94	1.67	11.08
Digestive system diseases	2.25	13.62	4.97	9	2.37	13.04	3.33	7.81
Infectious and parasitic diseases	7.13	7.14	2.18	6.82	5.28	7.53	1.43	7.39
Genitourinary system diseases	6.15	16.86	5.6	12.07	2.91	8.31	2.57	5.96
External causes	2.02	3.5	1.38	3.78	1.51	3.78	0.85	4.67
Endocrine, nutritional and metabolic diseases	3.66	10.33	3.31	10.02	1.66	5.57	1.24	4.57
Nervous system diseases	3.93	1.68	1.92	1.06	5.62	4.78	2.85	3.49
Mental and behavioral disorders	2.31	9.86	8.03	7.19	1.2	4.46	2.76	2.72
Neoplasms	1.08	6.73	1.28	7.61	0.4	2.15	0.54	2.44
Musculoskeletal system and connective tissue diseases	0.96	2.9	0.74	2.23	1.23	2.52	0.93	2.24
Other causes	0.79	3.5	1.03	3.51	0.63	2	0.65	1.8

Source: DATASUS, 2012^(8)^.

The hospitalization rates of the elderly, in general, were higher than in the population aged 20–59 years, being nine-fold higher regarding the circulatory system diseases; with the exception for mental and behavioral disorders, in the male population, and from the same cause besides genitourinary system diseases among women.

On table 4, one can further see the cost indexes (value spent per person) for each class of disease. Despite the fact that adults present with a greater number of hospital admissions (Table 1), individuals aged 60 years or more are responsible for an index of expenses with hospitalization that can be eightfold greater for circulatory system diseases. Except for mental and behavioral disorders in males, and for genitourinary system diseases in females, the expenses per person of the elderly are higher than those of the adult population (Table 4).

## DISCUSSION

Considering the use of a secondary database coming from healthcare services, such as the SIH-SUS, a few limitations can be identified: the possibility of emitting more than one AIH (hospital admission authorization) for the same individual (prolonged hospital length of stay, transfers among hospitals or readmissions), and the remunerative structure of the system that privileges financial logic in detriment of epidemiological logic, facts that can compromise information reliability and validity.

Nevertheless, such data have been increasingly more used in epidemiological research, besides the fact that such a system offers an expressive volume of data that represents about 80% of admissions of the entire hospital network of the country, factors that can reproduce such questioning^(6)^.

As to the data raised by the present investigation, hospitalizations in the adult population predominated, reaching 77% of hospitalizations among people of the female gender. As to the elderly population, one notes a prevalence of use of hospital services 1.6-fold greater in males, following the tendency of other studies^(6,9,10)^.

The value paid for hospitalizations is greater among the adult population, a reflection of the greater number of users of SUS at working age. However, the ratio of cost per inhabitant is expressively greater in the elderly population, especially among men, which enables stating that hospitalizations of the elderly are more costly than those that occur among individuals between 20 and 59 years of age. A study performed in 2004 found a cost ratio about four times higher among the elderly relative to the adults^(11)^.

Taking into consideration the differences between the genders, one must consider aspects inherent to the health culture of society, in which men, since early times, do not have the habit of caring for their health nor of seeking treatment at healthcare units. With time this fact leads to an increase of diseases in this population that are generally diagnosed at more advanced stages, requiring more specialized and expensive treatments. Whereas women tend to seek health care units more frequently, having treatments and investing in prevention and self-care, justifying less hospital expenses when they are aged.

The mean value of hospitalizations expressed the greatest cost in the Southeast Region, which was justified by longer hospital stay among its users, despite the fact that the daily cost of admissions in the North and South Regions has higher values. No references were found in literature covering the distribution of diseases with a national perspective, but other studies about specific disorders, such as suicide^(6)^ and genitourinary system diseases^(12)^, described higher expenses with hospitalizations along the South-Southeast axis.

One must consider that the most prevalent diseases up until the beginning of this decade, i.e., circulatory, neurologic, and neoplastic diseases, had their mean values decreased relative to mental and behavioral disorders, and musculoskeletal conditions. This is considered as a possible result of health-related actions performed by the Family Health Strategy (ESF, acronym in Portuguese), which has assisted the population in promoting health, control and prevention of diseases.

Such actions have been the focus of recent studies that considered the ESF a protective factor for the elderly as to chronic and degenerative diseases^(13)^, in addition to specific approaches as to stroke^(14)^, other cerebrovascular and cardiovascular events^(15)^, and circulatory system diseases^(16)^, which in this case have an extensive relation with vaccination of the elderly against the influenza virus, as a national strategy focused on the Primary Care services as from 1999.

In considering the higher results of the mean value of hospitalization and hospital length of stay, there is an expressive amount of cases related to mental and behavioral disorders. One of the primary etiologies, which may be associated to this result, is *delirium*, an acute organic ailment of which onset will initiate a series of events that can culminate with loss of independence, increased morbidity and mortality, besides elevating length and costs of hospitalization and care after hospital discharge^(17)^.

As to musculoskeletal and connective tissue diseases, those with greatest prevalence were osteoporosis, arthrosis, chronic spondylitis, arthritis, and rheumatism, with growing numbers and consolidating as a public health concern^(18)^.

In calculations relative to the rate of hospital admission and cost indexes, the prevalence of circulatory system diseases was observed among adults. In a study that evaluated the diseases that most affected the elderly during the period from 1998 to 2005 in a city in the state of Paraná^(18)^, a predominance of cardiovascular disorders was also observed, as well as in a study that analyzed national information during two specific periods: 1994 and 2005, with 32% and 28% of cardiovascular events, respectively^(19)^.

A prospective study performed in Greece comparing hospital admissions in three distinct months of the same year identified similar results, especially regarding the greater prevalence of hospitalizations due to circulatory system diseases, followed by musculoskeletal and gastrointestinal diseases^(20)^.

As to hospitalizations of the elderly related to the respiratory system, a large part presents with a direct relation with the influenza virus, which in Brazil has a seasonal distribution and an approximate prevalence of 22 cases per thousand inhabitants, influenced by geographic density, age distribution of the population, and climate conditions. Complications of this virus may cause severe respiratory problems, such as pneumonia^(21)^.

In addition to a better perception and evaluation of the strategies of Primary Care, especially within the ESF context, the role of health professionals, especially nurses, overcomes difficulties, such as lack of human and material resources, training of professionals, and an adequate physical structure, prioritized by care aligned with human values, respect, and autonomy for the elderly person, which may contribute favorably towards reduction of the most common diseases in the aged^(21)^.

In a broader context of health promotion, the inclusion of the elderly in activities that favor their social interaction, life habits, etc, should be highlighted. Some initiatives, such as a group of elderly individuals linked to a hospital geriatrics service, identified improved overall quality of life in the individuals, resulting from the psychological and social support they received from peers and professionals, and the benefits from the group activities, as well as a tendency towards elevation observed in the social relation and general domains^(22)^.

A public initiative that may be effective in most frequent diseases of the elderly and in longer hospital stays, is the *Academia da Cidade* (City Gymnasium)^(23)^ implemented by the Ministry of Health, in 2011. This initiative seeks to create places where people can exercise, engage in sociocultural activities, receive orientation, and develop projects integrated with Primary Care, which could serve even as an entry into the health system. Some studies^(24)^ have already shown the effectiveness of this proposal, which is not only for the elderly, but they generally are the ones that participate in it, in addition to those who would not have the means of paying to go to a gymnasium.

As a consequence of an older population, health promotion (in the vastness of its practice), actions of health education, prevention of diseases, and retarding of diseases and fragilities, as well as maintenance of independence and autonomy, may guarantee better quality of life to the elderly.

## CONCLUSION

In the present investigation, distinct indicators were used to evaluate costs related to hospitalization of the elderly. Without going into the merit of the scope of each one, the results showed differences and similarities, composing a more complete picture for assessing of such health costs.

The results presented here allow one to infer that admissions of the elderly population are more costly than those conditioned by the adult population, expressed by most of the indicators observed. Health complications related to the aging process itself are covered, such as chronic diseases, possible failures in health promotion actions and prevention of diseases, in addition to an increment in prevalence of diseases rarely investigated in literature to date, such as the primary causes of the expenses.

These effects may be reduced with the adoption of more comprehensive policies and greater investment in actions of promotion, prevention, and opportune and adequate treatment for the most prevalent diseases in the aged. These policies may and should serve as subsidies for the preparation and implementation of actions capable of bringing favorable changes to the lives of the elderly and to the Unified Healthcare System, as the structure in charge of their care, among many other responsibilities.
